# A lab-scale biofiltration system for mitigating diluted methane emissions

**DOI:** 10.1016/j.ohx.2026.e00755

**Published:** 2026-03-01

**Authors:** Tate Geiger, Camila González, Johannes Ali, Juliana Vasco-Correa

**Affiliations:** Department of Agricultural and Biological Engineering, The Pennsylvania State University, 436 Shortlidge Rd, University Park, PA 16802, United States

**Keywords:** Bioreactor, Packed bed, Biofilter, Greenhouse gases

## Abstract

Methane (CH_4_) is a potent greenhouse gas with a global warming potential 27–30 times greater than CO_2_. Diffuse sources such as livestock facilities, landfills, and coal mines emit methane at low concentrations (<5% v/v), limiting the applicability of conventional mitigation technologies. Biofiltration, which uses methanotrophic bacteria to oxidize CH_4_ to CO_2_, offers a sustainable alternative but lacks standardized hardware, resulting in inconsistent designs and limited reproducibility. We present an open-source, modular lab-scale biofiltration system optimized for continuous operation and experimental replication. The system comprises triplicate packed-bed columns constructed from chemically resistant materials, integrated with mass flow controllers for precise gas delivery, humidifiers to maintain moisture, and standardized fittings for leak-free assembly. Its transparent columns enable visual monitoring, and the bottom-up flow design minimizes media compaction. Validation at an inlet concentration of 0.5% CH_4_ achieved mean removal efficiencies of 89.0 ± 6.7%. Design files, bill of materials, and assembly instructions are provided under a CERN OHL license to facilitate adoption and customization. This hardware supports methane mitigation research and broader applications in gas-phase bioprocessing, enabling reproducible studies and accelerating development of scalable biofiltration technologies.

## Highlights

• Open-source modular lab-scale biofiltration system for methane mitigation research.
• Triplicate packed-bed bioreactor columns enable reproducible laboratory studies.
• Modular biofiltration system easily scales for multiple experimental setups.
• Complete design files and bill of materials provided for rapid customization.

## Graphical abstract

**Figure:**
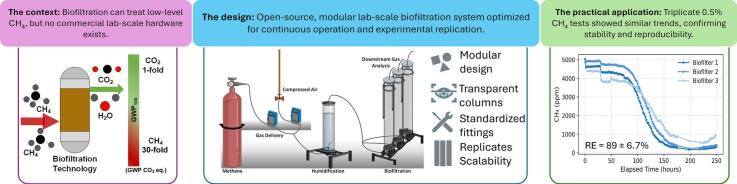

Specifications table

**Table:** 

Hardware name	Lab-scale biofiltration system for mitigating diluted methane emissions
Subject area	Environmental, planetary and agricultural sciences
Hardware type	Other: Bioreactor
Closest commercial analog	No commercial analog is available
Open source license	CERN OHL
Cost of hardware	5,450 USD (2,380 USD excluding mass flow controllers)
Source file repository	http://doi.org/10.17632/wnxvz3br5w.2

## 1 Hardware in context

Methane (CH_4_) is a potent greenhouse gas with a 100-year global warming potential (GWP_100_) approximately 27–30 times greater than that of carbon dioxide (CO_2_) [1]. A significant portion of anthropogenic CH_4_ emissions originates from diffuse sources such as landfills (16%), coal mines (6%), enteric fermentation (25%), and manure management (9%) [2], [3]. These sources often emit methane at low concentrations (<5% v/v), which limits the applicability of conventional capture, thermal, or catalytic treatment technologies. Mitigating these low-concentration emissions is critical to reducing their environmental impact and addressing climate change. In this context, biological methane oxidation via biofiltration emerges as a viable and sustainable strategy, particularly for emissions from livestock facilities and other diffuse sources of methane. Methane biofiltration is a multiphase, solid-state process in which microbial consortia, primarily methanotrophic bacteria derived from compost or soil, oxidize CH_4_ to CO_2_, substantially reducing its GWP [2], [4]. The efficiency of this microbial process depends on several environmental factors, including temperature, moisture, and nutrient availability [5].

Although microbial biofiltration has been widely applied for the treatment of ammonia and odorous compounds [6], [7], its application to methane is comparatively less developed. Laboratory-scale studies have explored biofiltration for methane mitigation from coal mines [8], landfills [9], [10], sewage treatment facilities [11], and livestock operations [12], [13], with reported removal efficiencies ranging from 13% to 99% [2]. This wide performance variability is attributed to empirical reactor designs, limited understanding of gas-phase mass transfer and biodegradation mechanisms, and significant inconsistencies in operational conditions.

Despite these advances, methane biofiltration systems are generally developed ad hoc in research laboratories, as there are no standardized, off-the-shelf solid-state bioreactors commercially available for gas-phase biological treatment. As a result, researchers are often required to fabricate their own packed-bed reactors, gas delivery systems, and instrumentation setups. This process is time-consuming, requires engineering expertise, and contributes to the heterogeneity of biofiltration system designs and experimental methodologies found in the literature. Although prior studies report biological performance and describe the media used, typically compost [14], [15], soil [16], woodchips [15], vermiculite [17], or expanded clay, they often omit detailed hardware specifications and operational guidelines. Nevertheless, some studies provide useful insights, particularly concerning the configuration of gas delivery systems [[18], [19]] and general column fabrication details, such as material and dimensions [[15], [18], [20]]. While these studies offer a valuable foundation that informed our design choices, critical aspects such as column construction, leak prevention, gas flow consistency, and the ability to replicate experiments across multiple units are rarely documented.

Comparable hardware used in other research areas, such as soil columns, lysimeters, or laboratory leach columns, is not designed for continuous gas-phase operation or methane oxidation. Equipment from vadose zone studies (e.g., SoilMoisture Equipment Corp., UGT, HZDR) and hydrological simulations (e.g., Sepor Inc.) are typically intended for liquid-phase or moisture-percolation studies. Likewise, air biofilters used in industrial ventilation for odor or ammonia control are not amenable to laboratory-scale replication or to testing multiple packing materials and conditions in parallel. These limitations have constrained the scalability and reproducibility of methane biofiltration research and hindered the ability to perform statistically robust comparisons across studies. While pilot-scale biofilters have demonstrated removal efficiencies of 60% to 95%, variations in design methodologies and performance metrics continue to limit direct benchmarking and generalization [[21], [22]].

To address these challenges, we developed an open-source, modular methane biofiltration system optimized for long-term, continuous operation and high experimental reproducibility. The system comprises replicated packed-bed columns constructed from chemically resistant materials and fitted with interchangeable headplates and instrumentation ports to support gas sampling, pressure monitoring, and humidifier integration. The design allows for simultaneous operation of triplicate columns and multiple experimental conditions, enabling statistically meaningful comparisons. Beyond methane oxidation, this modular system is broadly applicable to other porous media studies that require controlled gas-phase flow, including soil and atmosphere exchange experiments, composting, volatile organic compounds (VOC) and ammonia biofiltration, and evaluations of novel environmental bioreactor materials.

## 2 Hardware description

**Fig. 1:**
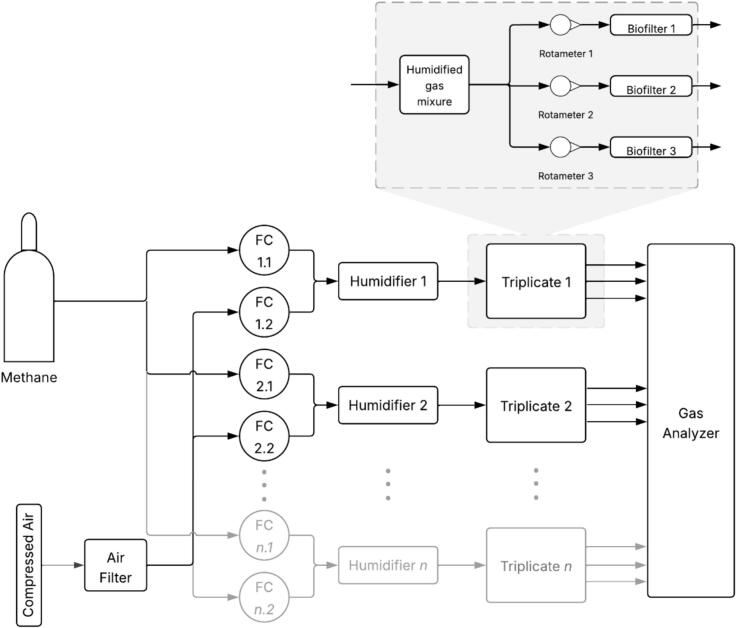
Diagram of modular lab-scale methane biofiltration setup. Methane and air flows are controlled using mass flow controllers (FC), mixed, and sent to humidifiers. Each saturated air-methane mixture is then split into three and sent to the triplicate biofilter columns. Gas mixtures exiting the biofilters are analyzed to determine their composition. This diagram illustrates the modular design of the system, which allows for the insertion of additional triplicate biofilter setups for simultaneous experimentation.

The laboratory-scale biofiltration system is designed as a modular, solid-state bioreactor for continuous methane oxidation under controlled conditions. Each unit consists of three transparent polycarbonate columns (triplicate biofilters) (Fig. 1) that are packed with porous media, enabling visual monitoring of media integrity and moisture distribution. The entire system is highly modular, allowing biofilter triplicates to be added as needed, and the columns can be run in parallel or series.

Gas delivery is precisely controlled using mass flow controllers rather than rotameters, ensuring accurate methane-air mixing and reproducible inlet concentrations. Integrated humidifiers maintain optimal moisture levels in the gas stream, limiting excessive drying of the solid support and thereby supporting microbial activity throughout extended operation (Fig. 1). These humidifiers are, in essence, shortened versions of the biofilter columns and use the same hardware and baseplate/headplate as the biofilters. The columns are sealed with machined acetal headplates and baseplates fitted with O-ring grooves for leak-proof assembly, while standardized push-to-connect fittings simplify setup and maintenance. The system employs a bottom-up gas flow configuration, which minimizes media compaction and facilitates drainage of excess water.

The design prioritizes accessibility and scalability; most components are 3D-printed, reducing fabrication cost and complexity, and only one part (the headplates/baseplates) requires conventional machining. The modular frame accommodates triplicate columns for statistical replication and can be easily expanded for additional experimental conditions (Fig. 1). The simplified lid design ensures a tight, leak-proof seal, and the design can be easily disassembled and cleaned between experiments.

This design is easily adaptable for other bioprocess and biofiltration systems that require solid-state packed-bed bioreactors, and the head/baseplates of the columns can be modified with additional ports for other applications. The design is thoroughly documented through open-source design files and assembly instructions, enabling researchers to replicate, customize, and adapt the system for methane mitigation or other gas-phase bioprocess applications. Though the scale we selected is typical for lab-scale methane biofiltration experiments, the design could also be easily scaled down, and we have provided parametric CAD for this purpose.

This hardware offers broad utility beyond methane biofiltration. Researchers in various fields can leverage its modular design and adaptability for the following purposes:

• Accelerates experimental design by providing a ready-to-use platform for testing gas-phase bioprocesses, reducing time spent on custom hardware fabrication.
• Enables comparative studies across different packing materials, microbial communities, or environmental conditions, supporting innovation in solid-state bioprocess research.
• Supports interdisciplinary applications, such as soil-atmosphere exchange modeling, compost aeration studies, and biofiltration of additional contaminants such as ammonia, VOC, and odors.
• Serves as a teaching and training tool for demonstrating principles of biofiltration, gas mixing, and microbial methane oxidation in academic settings.

## 3 Design files summary

The design files listed in Table 1 include all CAD files and 3D printing files for the biofilter head/baseplates, as well as frame feet, extrusion mount, and tube clamps to mount the biofilter in its support. Additionally, the files include a metal grate added inside the biofilter for solid media support and a manometer mount.

**Table 1:** Summary of design files.

**Design file name**	**File type**	**Open source license**	**Location of the file**
Headplate_Baseplate	STEP	CERN OHL	http://doi.org/10.17632/wnxvz3br5w.2
Headplate_Baseplate Machinist Drawing	PDF	CERN OHL	http://doi.org/10.17632/wnxvz3br5w.2
Frame Feet	STEP	CERN OHL	http://doi.org/10.17632/wnxvz3br5w.2
Frame Feet Print File	3MF	CERN OHL	http://doi.org/10.17632/wnxvz3br5w.2
Frame Feet Print File	STL	CERN OHL	http://doi.org/10.17632/wnxvz3br5w.2
Extrusion Mount	STEP	CERN OHL	http://doi.org/10.17632/wnxvz3br5w.2
Extrusion Mount Print File	3MF	CERN OHL	http://doi.org/10.17632/wnxvz3br5w.2
Extrusion Mount Print File	STL	CERN OHL	http://doi.org/10.17632/wnxvz3br5w.2
Biofilter Tube Clamp Left	STEP	CERN OHL	http://doi.org/10.17632/wnxvz3br5w.2
Biofilter Tube Clamp Right	STEP	CERN OHL	http://doi.org/10.17632/wnxvz3br5w.2
Biofilter Tube Clamp Print File	3MF	CERN OHL	http://doi.org/10.17632/wnxvz3br5w.2
Biofilter Tube Clamp Print File Left	STL	CERN OHL	http://doi.org/10.17632/wnxvz3br5w.2
Biofilter Tube Clamp Print File Right	STL	CERN OHL	http://doi.org/10.17632/wnxvz3br5w.2
Metal Grate (inch)	DXF	CERN OHL	http://doi.org/10.17632/wnxvz3br5w.2
Metal Grate (mm)	DXF	CERN OHL	http://doi.org/10.17632/wnxvz3br5w.2
Manometer Mount	STEP	CERN OHL	http://doi.org/10.17632/wnxvz3br5w.2
Manometer Mount Print File	3MF	CERN OHL	http://doi.org/10.17632/wnxvz3br5w.2
Manometer Mount Print File	STL	CERN OHL	http://doi.org/10.17632/wnxvz3br5w.2

### 3.1 Headplate/baseplate

The headplate and baseplate provide the seal for the top and bottom of the biofilter and humidifier columns. Design files, including CAD and shop drawings, are provided for machining.

### 3.2 Frame feet

These 3D-printed parts press-fit into the 2020 aluminum frame. Both STL and 3MF files are provided, and the latter include print settings.

### 3.3 Extrusion mount

The biofilter sits on this 3D-printed part, which bolts to the 2020 aluminum frame. Both STL and 3MF files are provided, and the latter include print settings.

### 3.4 Biofilter tube clamp

These two parts, which are mirror images of one another, clamp the outside of the biofilter column to provide a mounting point. CAD and STL files are provided for both the left and right tube clamp. 3MF files include both sides printed together with appropriate print settings.

### 3.5 Metal grate

These discs are laser cut from stainless steel and support the solid medium inside the biofilter column. DXF files are provided for laser-cutting services.

### 3.6 Manometer mount

This 3D-printed part secures the manometers to the biofilter column. Both STL and 3MF files are provided, and the latter include print settings.

## 4 Bill of materials summary

A detailed Bill of Materials (BOM) is provided in http://doi.org/10.17632/wnxvz3br5w.2 [23]. Efforts were made to include accurate and current pricing for all components in the BOM. However, these prices reflect U.S. costs at the time of publication and may vary substantially in other regions due to shipping, import duties, and local supplier availability.

The system incorporates several custom-designed 3D-printed components, including clamps, mounts, and support structures. These parts were originally printed using polylactic acid (PLA) filament on a Prusa i3 MK3S + printer (Prusa Research). However, the designs are compatible with most standard fused deposition modeling (FDM) printers and can be easily reprinted or modified based on user needs. The price of these parts was estimated based on the weight of the filament. Additional details, as well as the STL files and print settings, are included in the Supplementary Material [23].

The cost of gas analysis equipment, such as Fourier Transform Infrared (FTIR) analyzers or gas chromatographs, is not included, as it varies widely depending on the specific analytical requirements of the experiment. Similarly, prices for gas cylinders are excluded, as they depend on supplier availability and regional safety regulations.

## 5 Build instructions

### 5.1 System assembly and common hardware components

The biofiltration system was designed using a modular and standardized assembly approach to ensure consistent setup, simplify maintenance, and facilitate operation and reproducibility. Common hardware elements, such as tubing, fittings, headplates, and baseplates are standardized across all system modules, including the gas mixing, humidification, and biofiltration modules. This design strategy supports interchangeability between parts, enabling easy reconfiguration or replacement of system components.

#### 5.1.1 Modular design and connection interfaces

Connections between system modules were made using 1/4″ (6.35 mm) outer diameter (OD) polyurethane tubing (McMaster-Carr, 5648 K2), which was selected for its flexibility and chemical resistance. For high-pressure sections, polytetrafluoroethylene (PTFE) tubing (McMaster-Carr, 5239 K12) was used in combination with Yor-Lok fittings (McMaster-Carr, 5182 K111), providing enhanced pressure tolerance and secure mechanical connections. Tubing segments of various lengths connected the gas-handling units, humidifiers, and biofiltration columns.

All pneumatic connections, including flow splitting and inter-modular linkages, were made using compatible 1/4″ push-to-connect valves, splitters, and adapters, installed in accordance with manufacturer specifications to ensure leak-free operation. Sealing between components was achieved using adapters and connectors that enabled plastic-to-plastic seals. This design ensures tight and reusable connections without the need for additional sealing compounds.

#### 5.1.2 Headplate and baseplate design

**Fig. 2:**
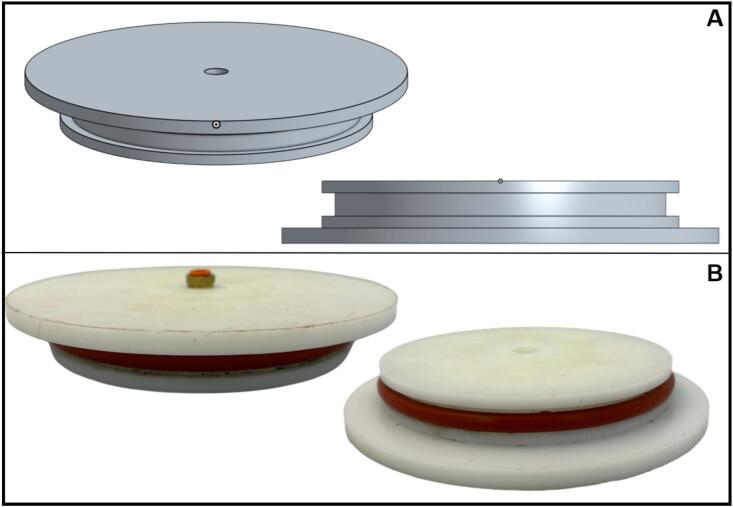
Design of headplates and baseplates for the biofilter columns and humidifiers. (A) CAD model and section view of the headplate and baseplate, showing the static piston seal groove design. (B) Machined components fabricated from acetal Delrin, assembled with a 30A silicone O-ring, showing a threaded push-to-connect fitting for gas lines.

The headplates and baseplates for the biofiltration columns and humidifiers were machined from food-safe acetal (Delrin, 7 in. diameter round stock; McMaster-Carr, 8572 K77). Each plate follows an identical design, making them interchangeable as a headplate or a baseplate (Fig. 2). Acetal was selected for its low cost, excellent machinability, dimensional stability, and resistance to moisture and chemical degradation.

Each plate was fitted with threaded bulkhead ports for gas inlets, outlets, and pressure relief valves. Threaded push-to-connect fittings (1/4″ NPT to 1/4″ OD tubing; McMaster-Carr, 7880 T125) were permanently installed in both the headplates and baseplates, providing fixed ports that enable quick, secure, and modular gas-line connections.

Sealing between the plates and columns was achieved using O-rings in a static piston seal configuration, designed according to the dimensional guidelines provided in the Parker O-Ring Handbook [24]. A single 30A durometer silicone O-ring (McMaster, 1173 N629), the softest available, was used to simplify lid installation and removal. The oversized groove supporting the O-ring facilitates easy cleaning and enhances the seal's reusability. Machining details are included in the Supplementary Material, which includes detailed descriptions, CAD files, and machinist drawings [23].

#### 5.1.3 Assembly and maintenance tools

Assembly and maintenance of the system require common laboratory and workshop hand tools. A tubing cutter (McMaster-Carr, 8288A51) was used to obtain clean, perpendicular cuts on the 1/4″ (6.35 mm) OD polyurethane tubing to ensure leak-free connections. Open-end wrenches and adjustable spanners were used to tighten compression and bulkhead fittings, while a set of hex keys and screwdrivers were used to secure column components, clamps, and manifold mounts. These readily available tools enable the system to be assembled, disassembled, and serviced without specialized equipment, thereby supporting reproducibility and facilitating ease of adoption in other laboratories.

### 5.2 Design and construction of biofiltration system modules

To facilitate understanding of the methane biofiltration system design, the design and construction details are presented in five sections, following the direction of gas flow through the system: i) gas delivery system, ii) humidifiers, iii) replicate flow distribution, and iv) biofilter columns.

#### 5.2.1 Gas delivery system

**Fig. 3:**
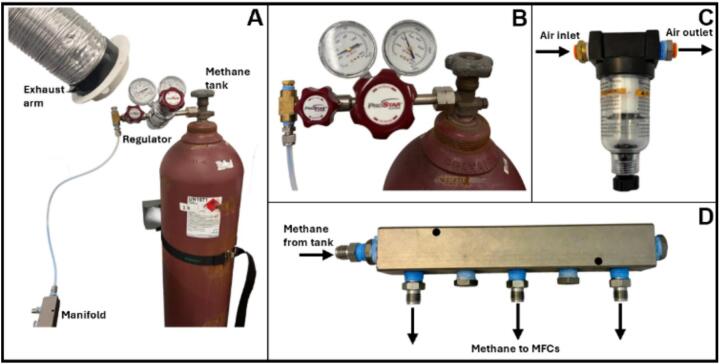
Gas delivery system used for air and methane supply in the biofiltration setup. (A) Overall system configuration for methane supply showing the methane cylinder with the required safety strap, pressure regulator, exhaust arm, and manifold connection. (B) Close-up of the pressure regulator connected to the methane tank. (C) Air filter used in the compressed air supply. (D) Stainless-steel manifold used to distribute methane from the cylinder to individual mass flow controllers (MFCs).

Air-methane gas mixtures were generated using compressed air from the laboratory facility and methane supplied from a certified high-purity gas cylinder. The methane cylinder was stored and operated following institutional and local safety regulations. The tank was secured with a safety strap to a fixed support and equipped with a pressure regulator (ProStar, 20123301350) and Yor-Lok fittings (McMaster-Carr, 5182 K111) to prevent accidental release of methane (Figs. 3A and 3B).

A local exhaust arm was installed near the regulator to capture any potential leaks (Fig. 3A), and a methane detection system was in place to continuously monitor gas concentrations in the surrounding area. These safety measures ensured full compliance with laboratory safety standards for the handling of flammable compressed gases.

The methane cylinder regulator was set to 40 PSI (276 kPa), as recommended by the manufacturer of the mass flow controllers (MFCs). In accordance with best practices for MFC operation, the compressed air supply was filtered using a 5-µm filter (McMaster-Carr, 4274 K94) before entering the MFCs (Fig. 3C). Methane from the cylinder was connected to a stainless-steel manifold (McMaster-Carr, 5975 K72) using 1/4″ OD (6.35 mm) PTFE tubing and a Yor-Lok stainless steel connector (Fig. 3D). The manifold was equipped with high-pressure Yor-Lok compression fittings and was used to divide the methane supply into individual supply lines, ensuring uniform and safe gas distribution upstream of the MFCs.

**Fig. 4:**
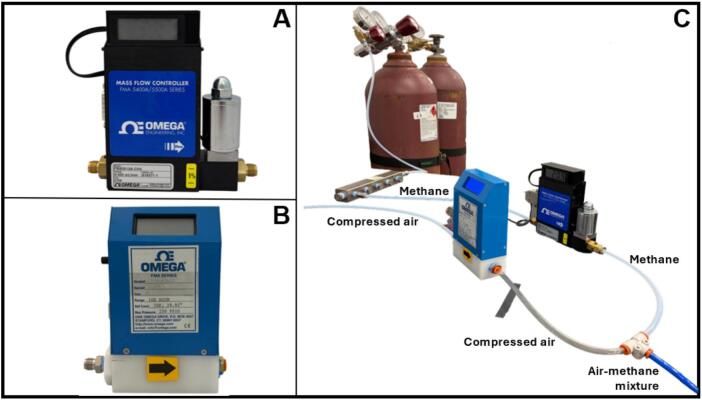
Mass flow controllers (MFCs) used for regulating air and methane flow rates in the biofiltration system. (A) Dwyer–Omega FMA-5500A series MFC. (B) Dwyer–Omega FMA-A2400 series MFC. (C) Experimental configuration showing the setup of MFCs for air-methane mixture control. Air and methane were supplied through independent MFCs connected in parallel and mixed using a push-to-connect T fitting to achieve the desired inlet methane concentrations.

To achieve the desired methane inlet concentrations, methane and air flow rates were regulated using Dwyer–Omega mass flow controllers (MFCs; models FMA-5508A, FMA-5510A, FMA-5512A, FMA-5518A, FMA-5524A, FMA-A2403, and FMA-A2409; Dwyer–Omega) (Figs. 4A and 4B). The FMA-5500A series MFCs required an external direct current (DC) power supply (model FMA545PW; Dwyer–Omega). All MFCs were equipped with 1/4″ NPT inlet and outlet ports. For most ports, 1/4″ NPT – 1/4″ push-to-connect were used, except for the high-pressure inlet of the methane MFCs, where 1/4″ NPT – 1/4″ Yor-Lok connectors were used to ensure a secure connection.

The MFCs were factory-calibrated for nitrogen (N_2_). To operate them with different gases, correction factors (*K-factors*) were applied in accordance with the manufacturer’s gas conversion specifications. The *K-factor* of air relative to N_2_ is 1.00, whereas the *K-factor* of methane relative to N_2_ is 0.72. Accordingly, the actual gas flow through each MFC was determined by applying the appropriate correction to the setpoint. For air, since the *K-factor* is identical to that of N_2_, the desired flow rate was entered directly in liters per minute (lpm). For methane, the corrected controller input ($Q_{CH_{4},\;corrected}$) was calculated as:

(1) $$Q_{CH_{4},corrected}=\frac{K_{N_{2}}}{K_{CH_{4}}}Q_{CH_{4},setpoint}$$

where $Q_{CH_{4},setpoint}$ is the target methane flow rate, expressed in standard cubic centimeters per minute (sccm). The corrected flow value was entered into the MFC controller to ensure accurate delivery of the intended methane concentration. The metered methane and air streams were combined downstream using 1/4″ push-to-connect tee fittings, allowing sufficient mixing along the connecting tubing between the MFCs and the humidification units (Fig. 4C). Preliminary tests confirmed that additional static mixing was unnecessary, as complete homogenization occurred within the tubing prior to gas humidification and distribution to the biofilter columns.

#### 5.2.2 Humidifiers

Successful biological methane oxidation in biofilters requires maintaining adequate moisture levels within the packing material to sustain microbial activity. To prevent the incoming gas phase from drying out the packing medium, the inlet air-methane mixtures must be humidified prior to entering the biofiltration columns. To achieve this, each of the air-methane mixtures, corresponding to a specific concentration, is humidified by bubbling the gas through a water column before being distributed to the biofilter columns. As a result, the system design requires one humidifier column per gas mixture concentration.

**Fig. 5:**
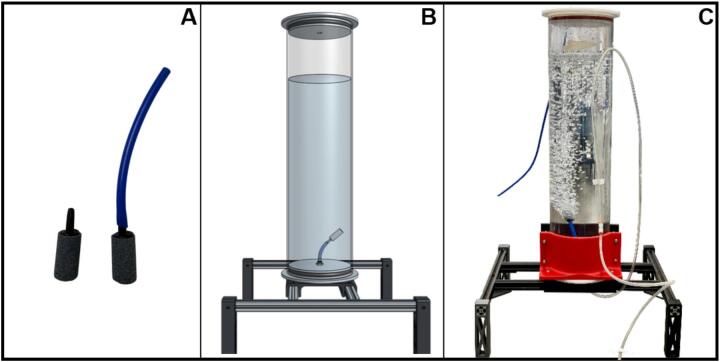
Humidifiers used in the biofiltration system for saturation of the inlet air-methane mixture. (A) Left: ceramic air diffuser. Right: ceramic air diffuser connected to a polyurethane tube segment used to attach the diffuser to the baseplate of the humidifier. (B) CAD representation of the humidifier. (C) Experimental setup of the humidifier.

The main body of each humidifier was constructed from polycarbonate tubing (McMaster-Carr, 8585 K58, 2 ft length), measuring 0.15 m in internal diameter and 0.61 m in height. Each column is filled with deionized water to a level of 0.50 m. The gas mixture enters the humidifier from the bottom of the column and exits from the top. A ceramic air diffuser (Pawfly, B075QCWGZQ), commonly known as an aquarium air stone, was placed at the gas inlet to produce fine bubbles. The diffuser was selected for its low cost, fine bubble dispersion, and ease of replacement (Fig. 5A). The diffuser was connected to a push-to-connect fitting (McMaster-Carr, 7880 T125) installed on the baseplate, using a short segment of polyurethane tubing. The tubing was friction-fitted to the diffuser’s barbed inlet, providing a secure, leak-free connection.

Figs. 5B and 5C show the CAD model developed for the humidifiers alongside the humidifier used in the laboratory setup. To prevent water backflow from the humidifier into the gas supply line, especially toward the upstream MFCs, a simple gravity-based loop was incorporated into the inlet tubing. Specifically, the gas line was routed upward above the body of the humidifier, then back down into the base of the humidifier. This configuration relies on gravitational pressure to overcome any water pressure at the diffuser, effectively blocking reverse flow into MFCs under static or low-flow conditions.

System performance was verified by connecting a closed chamber downstream of the humidifier and monitoring relative humidity using a digital temperature–humidity sensor (AcuRite, B0013BKDO8). The outlet air consistently exceeded the sensor’s upper calibration limit (95% relative humidity), confirming effective humidification.

#### 5.2.3 Flow distribution to biofilter replicates

The biofiltration system was designed to operate three replicates per experimental condition, enabling statistical evaluation of process performance and microbial variability under identical operational parameters. Running replicates in parallel under controlled gas flow and concentration conditions provides experimental reproducibility and statistical reliability of observed trends, while reducing random variation due to microbial behavior or flow-distribution inconsistencies.

**Fig. 6:**
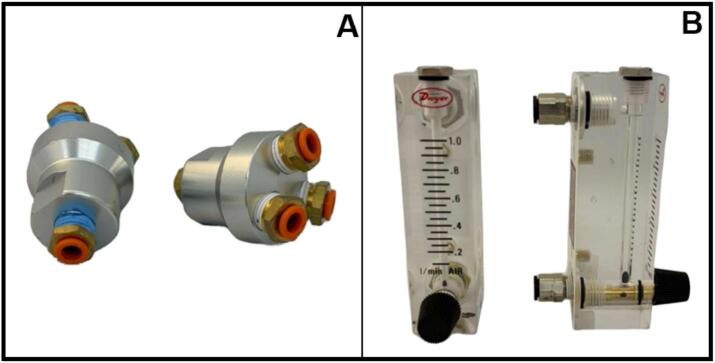
Components used for gas flow distribution to biofilter replicates. (A) Aluminum three-way manifold with 1/4″ push-to-connect fittings used to evenly divide the humidified air-methane mixture among the biofilter replicates. (B) Variable-area rotameter used for individual measurement and manual adjustment of the gas flow entering each biofiltration column.

To achieve this, each humidified air-methane mixture (corresponding to a specific inlet concentration) was evenly divided among three biofilter columns (triplicate). After leaving the humidifier, the gas stream was routed to a three-way aluminum manifold (McMaster-Carr, 5465 K71), which ensured uniform pressure distribution and consistent flow splitting (Fig. 6A). Each outlet from the manifold was connected to an individual rotameter (Dwyer, series VF) to allow independent measurement and adjustment of the flow rate entering each biofilter (Fig. 6B). During setup, the rotameters were manually adjusted to ensure equal inlet flow conditions across the three replicates.

All gas lines were connected using 1/4″ (6.35 mm) OD polyurethane tubing and push-to-connect fittings installed on the manifolds, rotameters, and biofilter inlets to facilitate leak-free operation, as well as rapid assembly and maintenance.

#### 5.2.4 Biofiltration columns

**Fig. 7:**
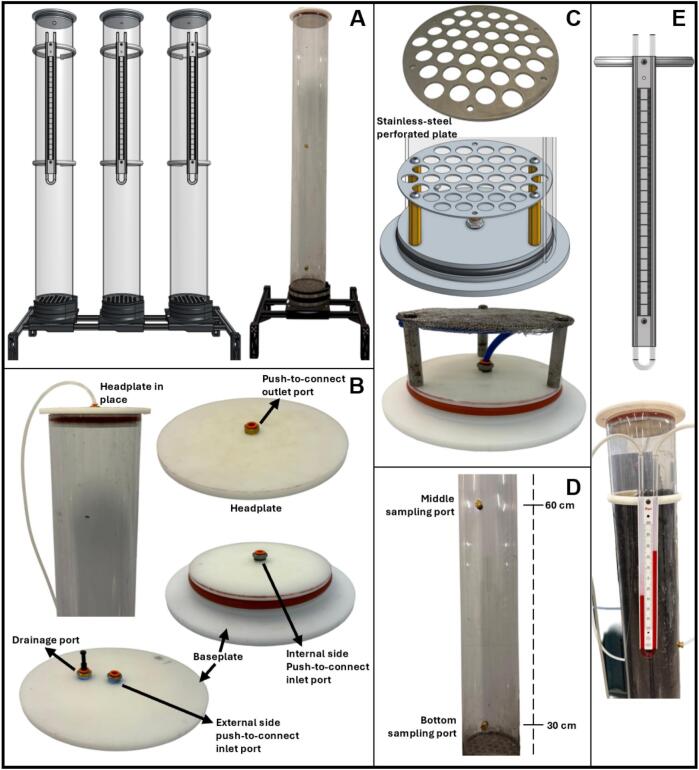
Components and assembly of the biofiltration columns. (A) Left: CAD model of a triplicate biofilter assembly mounted on the support frame. Right: Single biofilter column. (B) Biofilter baseplate and headplate showing inlet and outlet push-to-connect fittings and O-ring sealing. (C) Packing support assembly consisting of a stainless-steel perforated plate covered with stainless-steel wire cloth, mounted on four 50 mm hex standoffs resting on the baseplate, forming a 6 cm plenum beneath the packing material. (D) Column wall showing the position of sampling ports relative to the baseplate. (E) U-tube manometers connected to the inlet and outlet ports for pressure measurement along the biofilter column.

The main body of each column was constructed from polycarbonate tubing (McMaster-Carr, 8585 K58, 4 ft length), measuring 0.15 m in internal diameter and 1.22 m in height. Polycarbonate was selected for its high strength-to-weight ratio and optical transparency, which allows visual inspection of the packing material and gas–liquid interactions during operation. Both ends of each column were sealed with identical acetal (Delrin) headplates and baseplates, as described in Section 5.1.2 (Fig. 7A).

The gas inlet is located on the baseplate, where the air-methane mixture flows upward through the packed bed and exits via an outlet port on the headplate. The inlet assembly includes 1/4″ NPT push-to-connect fittings on both the external and internal sides of the baseplate. The external (underside) fitting connects to the humidifier outlet, while the internal fitting is linked to a short tubing segment that directs gas to the media support plate. This segment ensures that, if standing water accumulates in the plenum, the gas inlet remains above the waterline, preventing backflow into the supply tubing. The gas outlet on the headplate consists of a 1/4″ NPT port fitted with a push-to-connect fitting, to which a polyurethane tube is attached for downstream gas composition analysis (Fig. 7B).

The packing support was constructed using a 1/16″ (1.59 mm) thick stainless-steel perforated plate covered with 15 x 15 mesh stainless-steel wire cloth (McMaster-Carr, 85385 T62). This assembly is mounted on four 50 mm hex standoffs (McMaster-Carr, 94868A767), which rest on the baseplate (Fig. 7C). The wire cloth is secured to the plate by M5 bolts that pass through the wire cloth and plate before being fastened to the hex standoffs. The support structure creates a 6 cm plenum beneath the packing material, promoting uniform gas distribution by equalizing pressure and minimizing channeling. It also provides space for excess water accumulation, preventing contact with the packed bed. While not permanently fixed, the friction between the wire cloth and the polycarbonate wall holds the support in place, even when the column is inverted for media removal. An auxiliary drainage port is included in the baseplate to remove water that may accumulate in the plenum. This port remains sealed during normal operation but can be manually opened as needed (Fig. 7B). If frequent drainage is expected, a small tubing line and ball valve can be added to facilitate drainage.

Each column was packed with organic media, forming a 1-m-tall packed bed. The remaining column height (∼22 cm) served as a headspace for gas homogenization and sampling. To allow sampling of the packing material, the headplate can be removed, providing direct access to the top of the packed bed. Additionally, two threaded sampling ports were installed on the polycarbonate sidewall, 20 cm and 70 cm above the baseplate, respectively (Fig. 7D). These ports remained sealed with 1/4″ NPT threaded plugs during operation and were only opened during sampling. Packing material samples can be collected using a sterile plastic V-scoop spatula (Corning, 3006), ensuring minimal contamination and easy access through the ports.

To measure the pressure drop across the packed bed, U-tube manometers (Dwyer, 1221–8-D) were installed on each column. One side of each manometer was connected to the gas inlet and the other to the gas outlet using polyurethane tubing. Y-shaped push-to-connect fittings (McMaster-Carr, 1901 K109) were used to split the gas streams without interrupting flow (Fig. 7E). A custom 3D-printed clamp was designed to hold each manometer in place. A segment of pneumatic tubing was inserted into the clamp interior to enhance grip. An M5 nut was embedded in a nut trap, and an M5 bolt was used to tighten the clamp securely. The manometer was fastened to the clamp using a 15 mm long M5 bolt and nut (Fig. 7E).

#### 5.2.5 Biofilter support structure and mounting system

**Fig. 8:**
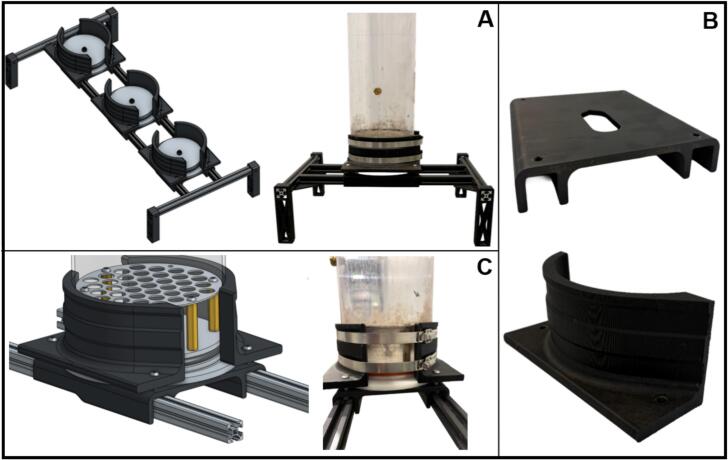
Support structure and mounting system used to secure the biofilter columns. (A) Left: Overall view of the aluminum extrusion frame supporting three biofilters. Right: Corresponding laboratory assembly for a single biofiltration column. (B) Top: 3D-printed PLA-based mount used to align and support extrusions during assembly. Bottom: 3D-printed clamps. (C) Close-up of mounting system setup. Left: CAD model of the mounting system. Right: Corresponding laboratory mounting system for a single biofiltration column.

To ensure stability, ease of assembly and scalability, a custom modular support frame was designed and fabricated in-house (Fig. 8A). Each set of biofilters was mounted on a structure built from 400 mm and 1000 mm lengths of 2020 aluminum extrusion (SeekLiny, B0B2P434PD). These widely available extrusions are offered in various lengths, enabling assembly without the need for machining or welding. The frame was designed to be fully modular and easily reconfigurable.

The frame consists of two horizontal rails supporting a series of 3D-printed PLA mounting bases, each holding one biofilter column (Fig. 8B). The short extrusions on each side connect to the long extrusions holding the biofilters via two L-brackets (SeekLiny, B09DYJX8YK). The frame stands on 3D-printed PLA feet, which can be press-fit or bolted onto the short extrusion. The feet were mechanically tested to ensure structural safety. Each column rests on its dedicated mount (Fig. 8B and 8C), which also serves as a spacer to align the extrusions during assembly.

To secure the biofilters, a clamp assembly was developed using two stainless-steel hose clamps (McMaster-Carr, 5416 K38). The hose clamps tighten the 3D-printed clamps around the polycarbonate column, providing a uniform force. The 3D-printed clamps are fixed to the aluminum frame with M3 bolts and T-nuts (POLISI3D, B07V1HX9GS) inserted into the extrusion channels. This configuration enables quick assembly and disassembly without the need for specialized tools (Fig. 8C).

### 5.3 Gas measuring system

The biofiltration system was not specifically engineered for a particular gas analyzer. Instead, this design offers flexibility in selecting analytical methods based on specific experimental requirements, including the quantification of methane, carbon dioxide, and other gases of interest. Each biofilter outlet terminates in a 1/4″ push-to-connect fitting on the headplate, enabling straightforward integration with either automated multipoint sampling systems or manual collection setups.

In automated operation, the outlet lines can be routed to a gas multipoint sampler, which sequentially directs samples from each biofilter to a central analyzer. This configuration accommodates instruments such as Fourier Transform Infrared (FTIR) analyzers, photoacoustic analyzers (e.g., Innova), or other real-time gas sensors for continuous monitoring of methane, carbon dioxide, or additional target compounds.

Alternatively, for offline analysis, the outlet ports can be used to fill gas bags or similar sampling vessels. These samples can then be analyzed using gas chromatography or any other laboratory-based analytical platform.

## 6 Operation instructions

### 6.1 Flow setup

Secure the methane gas cylinder by mounting it to a wall or fixed support using appropriate restraints to prevent accidental tipping or movement during operation (Fig. 3A). Attach a suitable pressure regulator directly to the tank outlet and adjust the outlet pressure as required (Fig. 3B). Identify the compressed air source and install the appropriate air filter to ensure a clean gas supply to the system (Fig. 3C).

Once both air and methane sources are established, connect the MFCs using the appropriate tubing and fittings (Fig. 4). The individual MFC outlets should be joined using a suitable tee fitting to form the mixed gas stream (Fig. 4C). All connections should be checked for leaks before operation. The soap bubble method can be used as a simple and effective leak-checking technique.

Before setting the MFCs' flow rates, the total gas flow must be calculated based on the number of biofilter columns, the desired flow rate per column, and the target methane concentration in the air stream. To determine the correct methane flow rate, the *K-factor* correction must be applied using Equation (1), as described in Section 5.2.1.

Once the corrected flow rates are known, the methane and air MFCs are adjusted to their respective setpoints using the built-in control knobs. After allowing the system to stabilize, the resulting gas mixture should be verified using a gas chromatograph or other available gas analyzer, depending on the analytical methods in use. If the measured methane concentration does not match the target value, the methane MFC setpoint should be fine-tuned incrementally until the desired concentration is reached. This approach ensures accurate and reproducible gas delivery to all biofilter columns.

### 6.2 Humidifier setup

Direct the mixed air stream toward the inlet port located on the baseplate of the humidifier. Ensure that the ceramic diffuser (Fig. 5A) is securely installed in the baseplate prior to operation. Fill the humidifier column with deionized (DI) water to approximately 50 cm in height (Figs. 5B and 5C), to maintain full humidification of the gas mixture. Over the course of the experiment, the water level gradually decreases due to evaporation and gas flow. It should be checked periodically and refilled as needed, typically every few weeks, depending on operating conditions.

After humidification, the air-methane stream should be split into the appropriate number of outlet lines using a manifold (Fig. 6A), corresponding to the number of biofilter replicates operating under each gas concentration. This ensures even distribution and consistent inlet conditions across all biofiltration columns.

### 6.3 Biofiltration columns setup

The preparation of the biofilter media begins with determining the moisture content of the packing materials selected for the experiment. Moisture content is measured using a gravimetric method. Representative samples of each material are weighed and dried in an oven at 105 °C until a constant weight is achieved, allowing calculation of the initial moisture content on a wet basis. Based on these values, the material is brought to the target moisture content by gradually adding deionized water while mixing thoroughly to ensure uniform distribution. Once conditioned, the media is added to the biofilter columns by hand in successive layers, gently settling each layer to avoid excessive compaction. This process continues until the predefined packed bed height is reached.

After the packing height is standardized across all columns, rotameters (Fig. 6B) are connected to the gas inlets at the baseplate of each biofilter and to the corresponding gas streams from the humidifiers. Because slight variations in flow resistance may exist between biofilters, the rotameters for each set of triplicates are adjusted to ensure that the same flow rate is achieved across all replicates.

Next, the supports for the manometers are installed, and the manometers are mounted on each column (Fig. 7E). Both the inlet and outlet tubing are split into two streams using Y fittings. One branch of the outlet tubing is connected to one side of the manometer, and one branch of the inlet tubing is connected to the other side of the manometer. This configuration allows for accurate pressure drop monitoring across each biofilter column. Finally, the column headplates are installed (Fig. 7B). A small amount of water can be used as a lubricant to facilitate O-ring insertion and ensure a proper seal during installation.

### 6.4 Gas monitoring

Accurate and continuous monitoring of exhaust gases is essential for evaluating methane oxidation performance and detecting byproducts such as carbon dioxide, nitrous oxide, or other target gases, depending on the experimental design and research objectives.

As described in Section 5.3, the biofiltration system is not built around any specific gas analyzer. Instead, the design supports full flexibility in selecting and configuring downstream gas monitoring setups, making it adaptable to a wide range of analytical tools and experimental requirements.

In this setup, gas monitoring was performed using both automated and manual approaches. Automated monitoring with FTIR technology enabled continuous data acquisition under stable operating conditions, while manual sampling provided independent validation of gas concentrations via gas chromatography. This complementary strategy ensured reliable measurements, enhanced data accuracy, and allowed for cross-validation between methods. Further details on both approaches are provided in the Supplementary Material [23].

### 6.5 Sampling of biofilter material

To evaluate spatial and temporal variations in microbial activity and physicochemical conditions within the biofilters, samples of packing material can be collected from both the side ports (Fig. 7D) and the top surface of each column. The side ports are strategically positioned along the column height to provide access to distinct depth zones (lower, middle, and upper layers) without disturbing the overall media structure.

Prior to sampling, gas flow to the corresponding biofilter must be temporarily interrupted to prevent backflow and avoid disruption of the packing material. Samples are withdrawn through the side ports using sterile spatulas, ensuring that the tool reaches the center of the column for a representative collection. Ports must be resealed immediately after sampling to maintain airtight conditions. Additional material can be sampled from the top layer by removing the column headplate and collecting media from the uppermost 2–3 cm of the bed using sterile tools. All collected samples are transferred to sterile containers and appropriately stored for downstream analysis.

### 6.6 End of experiment procedure

At the conclusion of each experimental run, the system must be safely shut down. The methane supply valve should be closed, and both the methane and air MFCs should be turned off to halt gas delivery. The compressed air line must also be closed to prevent backflow, and any residual gas in the tubing should be vented to atmospheric pressure through the exhaust line. Once the system is fully depressurized, the lids of both the humidifiers and biofiltration columns may be carefully removed to release any remaining internal pressure and to allow safe access to internal components.

Following depressurization, the packing material should be removed from each biofilter column. All tubing, ports, and fittings must be thoroughly cleaned and inspected after disassembly to eliminate microbial buildup or residue accumulation. The humidifier columns should be emptied, rinsed with deionized water, and dried prior to storage. The methane cylinder valve must be securely closed, and any residual pressure in the regulator should be safely vented. The regulator may be removed if long-term storage is anticipated.

This end-of-experiment procedure ensures a safe shutdown of the system and supports the collection of high-quality samples for post-experimental analysis.

### 6.7 Additional operational and safety recommendations

The following recommendations support safe, consistent operation and reflect key considerations identified during system setup and routine use.

• Tubing and fittings inspection: Before initiating operation, verify that all tubing and push-to-connect fittings, especially those connected to the humidifiers, are airtight. Use a bubble leak detection method to confirm sealing integrity prior to introducing pressurized gas.
• Tube preparation and O-ring protection: Before assembling the biofilters, prepare the polycarbonate tubes by deburring all edges and wiping the surfaces of the tube. Wipe interior and exterior surfaces with a lint-free towel. To facilitate the insertion of soft O-rings and prevent damage during assembly, the inner edge of each polycarbonate tube must be chamfered. Chamfering is performed using a manual deburring tool, with the chamfer angle set as steep as practically possible to minimize the risk of cutting or deforming the O-rings during installation.
• Sealing and plate installation: Due to the tight tolerance of the O-ring sealing design, additional leverage may be required to properly seat the headplates and baseplates. A stool or platform can be used to reach the top of the biofilter, to press the headplates securely into place without damaging components.
• Leak testing: Leak-proofing can be confirmed using a bubble leak test rather than a pressure-decay test, which is unsuitable due to the large surface area of the lids and risk of unseating under pressure. A soap solution is applied around the seals and threaded fittings to detect any leaks. Replace fitting or O-rings if any leaks are detected.
• Water quality considerations: Use only DI water in humidifier columns. If the local water supply contains high levels of dissolved minerals, it can cause scale buildup and clog ceramic diffusers. Even with DI water, periodic replacement of the diffuser may be necessary due to the gradual accumulation of minerals and potential flow restriction.
• Failsafe pressure release mechanism: A passive safety mechanism is integrated into the mounting system of the columns by sandwiching the baseplate between two 3D-printed support components. In the event of over-pressurization, the headplate is designed to detach first, reducing the risk of structural damage or baseplate ejection. During assembly, hose clamps securing the columns should be tightened before the frame bolts to minimize mechanical stress and improve system rigidity.

## 7 Validation and characterization

### 7.1 Methodology

To validate the performance and reliability of the biofiltration system, the assembled columns, gas delivery components, and measurement hardware were tested under controlled operating conditions. A constant flow rate of 1 L/min per column was maintained, with the target inlet methane concentration ($C_{CH_{4},inlet}$) set at 0.5% v/v (5000 ppmv). Methane levels were regulated using MFCs, and the air-methane gas stream was evenly split to supply three replicate columns with identical flow rates and concentration levels. Each column was packed with a 50:50 (dry weight basis) mixture of finished compost and wood chips, preconditioned to a standardized moisture content of 60% (wet weight basis) using DI water. Food and yard waste compost was obtained from the Penn State Organic Materials Processing Center (OMPEC), and woodchips were sourced from the Penn State Office of the Physical Plant Grounds Maintenance. The media was manually mixed in containers to achieve a homogeneous mixture and loaded into the columns to the specified bed height of 1 m.

System validation was carried out by continuously monitoring methane concentrations in the outlet gas from each column. Gas analysis was performed using a CAI FTIR gas analyzer (California Analytical Instruments, Model 600 Series), coupled with a CAI 700 Multipoint Sampler. A CAI Zero Air Generator supplied purified air for background correction and calibration. Each biofilter outlet was sampled sequentially for 15 min under identical environmental conditions. Between samples, the system was flushed with compressed room air to minimize cross-contamination. Spectral acquisition and compound quantification were performed using OPUS software, which identified gases based on compound-specific absorption peaks. This configuration enabled the simultaneous operation of the three biofilters under uniform conditions, ensuring accurate performance validation and allowing for a consistent assessment across all three biofilters.

To evaluate biofilter performance, we used methane removal efficiency (RE) and methane elimination capacity (EC), commonly applied metrics in biological gas treatment that measure how effectively the reactor eliminates CH_4_ from the incoming air stream. Methane removal efficiency (RE) compares the inlet and outlet methane concentrations, representing the proportion of CH_4_ oxidized within the column and it is calculated as:

(2) $$\mathrm{RE}\left(\%\right)=\frac{C_{CH_{4},in}-C_{CH_{4},out}}{C_{CH_{4},in}}\text{x}\;100$$

where $C_{CH_{4},in}$ and $C_{CH_{4},out}$ are the inlet and outlet CH_4_ concentrations, respectively.

Methane elimination capacity (EC) quantifies the mass of methane degraded per unit of reactor volume and time. It reflects the actual treatment rate of the system and is calculated as:

(3) $$\mathrm{EC}\left(\%\right)=\frac{{Q(C}_{CH_{4},in}-C_{CH_{4},out})}{V_{\mathrm{bed}}}$$

where Q and $V_{\mathrm{bed}}$ are the flow rate and packed bed volume, respectively.

Finally, biological replicate reproducibility was assessed by calculating coefficients of variation (CV) for outlet methane concentrations over time.

### 7.2 Results and discussion

**Fig. 9:**
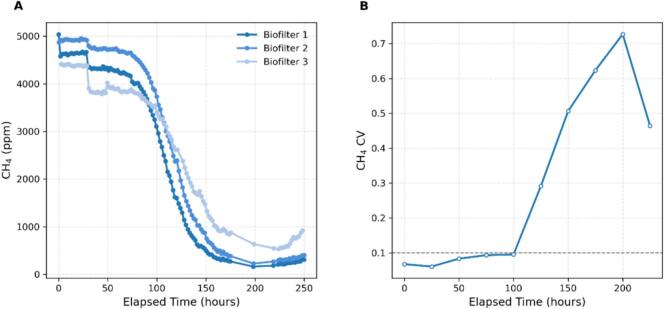
(a) Methane concentration profiles over time for three biofiltration columns operated under identical conditions. Inlet methane concentration of 5000 ppmv, air flow of 1 L/min, biofilters packed with a 50:50 (dry weight basis) mixture of industrial compost and wood chips, adjusted to an initial moisture of 60% (wet weight basis). Data represent the gas-phase CH_4_ concentration (in ppmv) measured at the column’s outlet. (b) Coefficient of variation (CV) of outlet CH_4_ concentration among the three biological replicates. The CV was calculated at each time point based on the mean and standard deviation of the three biofilters, providing a quantitative measure of reproducibility over time. The dashed line indicates a CV threshold of 0.1 for reference.

The temporal profiles of methane concentration at the biofilters outlet are shown in Fig. 9(A). All three columns exhibited a gradual decrease in methane levels during the first 150–180 h. After this initial transition period, the outlet concentration stabilized by the end of the experiment at 250 h. This stabilization corresponds to the establishment and metabolic activation of the methanotrophic community within the packing media. The resulting curves reflect the dynamic response of the biofilters and validate the reproducibility and stability of the experimental setup.

The final methane RE for the three biofilters were 93.7%, 91.9%, and 81.5%, respectively. The mean RE across replicates was 89.0 ± 6.7% (n = 3). The final EC for the three biofilters were 10.4 g m^-3^h^−1^, 10.21 g m^-3^h^−1^, and 9.05 g m^-3^h^−1^, respectively. The mean EC across replicates was 9.90 g m^-3^h^−1^ ± 0.74 g m^-3^h^−1^ (n = 3). The comparison of RE and EC across biological replicates explains the reproducibility of the system. While the final RE values showed noticeable variation among biofilters, the corresponding EC values were more closely grouped. This indicates that differences among replicates were more pronounced when expressed as relative removal efficiency than when expressed as volumetric elimination capacity while EC reflects the overall degradation rate normalized to airflow and reactor volume, resulting in lower relative dispersion among replicates. These results suggest that, despite some variability in residual methane levels, the three biofilters achieved comparable overall treatment capacities, supporting the reproducibility of the experimental setup.

The coefficient of variation (CV) analysis among replicates in Fig. 9(B) provides a time-resolved assessment of experimental reproducibility. During the initial period, CV values remained low, indicating that the three columns responded similarly under identical operating conditions. As the outlet methane concentration decreased rapidly, CV increased, suggesting divergence among replicates during the transition toward steady operation. This behavior can be explained by biological and structural heterogeneity across columns, including differences in microbial acclimation, moisture distribution, packing heterogeneity, and local mass transfer conditions. In addition, because CV is normalized by the mean concentration, variability can appear amplified at low methane concentration levels. The higher residual methane observed in Biofilter 3 during the later stage contributed to the elevated CV, highlighting that replicate differences were most pronounced under low outlet concentration conditions. Overall, Fig. 9(B) indicates strong reproducibility during the initial response and a transient increase in variability during the stabilization period. Despite this increase, the obtained CV values remain within acceptable ranges for biological environmental treatment systems, where inherent biological variability is expected.

## Declaration of generative AI and AI-assisted technologies in the writing process

The authors acknowledge the use of generative AI tools, specifically GPT-5 and Claude 3.5 Sonnet (accessed in November 2025), to assist with grammar correction, sentence restructuring, and improving clarity and flow during the preparation of this manuscript. All AI-generated suggestions were critically reviewed, edited, and approved by the authors. The authors take full responsibility for the accuracy, integrity, and content of the final manuscript. No AI-generated content was used without full author review, and all substantive ideas, analyses, and conclusions are solely those of the authors.

## CRediT authorship contribution statement

**Tate Geiger:** Writing – original draft, Visualization, Methodology, Investigation. **Camila González:** Writing – review & editing, Writing – original draft, Visualization, Project administration, Investigation, Funding acquisition, Data curation, Conceptualization. **Johannes Ali:** Writing – original draft, Validation, Data curation. **Juliana Vasco-Correa:** Writing – review & editing, Supervision, Project administration, Funding acquisition, Conceptualization.

## Declaration of competing interest

The authors declare that they have no known competing financial interests or personal relationships that could have appeared to influence the work reported in this paper.
